# Assessing inter‐observer variability in prostate and GTV segmentation on mpMRI: A comparison between radiation oncologists and AI‐based method

**DOI:** 10.1002/acm2.70563

**Published:** 2026-04-03

**Authors:** Philippe Dionne, André‐Guy Martin, Étienne Ouellet, Jean‐Christophe Roy, Ingrid Sidibé, Marie‐Anne Froment, Éric Vigneault, William Foster, François Bachand, Louis Archambault, Éric Poulin

**Affiliations:** ^1^ Département de physique, génie physique et d'optique, Faculté des sciences et de génie Université Laval Québec Canada; ^2^ Service de radio‐oncologie CHU de Québec ‐ Université Laval Québec Canada; ^3^ Axe Oncologie Centre de recherche du CHU de Québec ‐ Université Laval Québec Canada; ^4^ Service de radiologie CHU de Québec ‐ Université Laval Québec Canada

**Keywords:** mpMRI, segmentation, prostate cancer

## Abstract

**Background:**

Accurate delineation of the prostate and intraprostatic gross tumor volume (GTV) on multiparametric MRI (mpMRI) is critical for radiation therapy planning, particularly for focal dose escalation strategies. However, interpretation of mpMRI can be challenging and prone to inter‐observer variability, especially among radiation oncologists (ROs) who may have limited training in prostate MRI interpretation. In addition, because many patients do not undergo diagnostic mpMRI before treatment, radiologist input is often absent during treatment planning, which can compromise accurate GTV delineation.

**Purpose:**

To evaluate approaches for improving focal prostate GTV delineation on mpMRI for radiotherapy planning, by assessing the impact of radiology reports on ROs contours, and comparing the performance of an automated segmentation tool (AIRC) with radiologists' contours as a potential replacement when radiologist input is unavailable.

**Methods:**

Twenty patients underwent mpMRI at our institution. Delineation of the prostate gland and GTV was performed by ROs in two phases. In the first phase, ROs segmented both structures with access only to biopsy results, indicating positive core locations. In the second phase, they repeated the segmentation with access to the radiology report. AIRC segmented both structures as well, using the same images as made available to the ROs. Lesion detection performance was evaluated using sensitivity and positive predictive value (PPV). Segmentations were compared to consensus contours from two radiologists using the STAPLE algorithm. Similarity metrics computed included Dice similarity coefficient (DSC), Hausdorff distance (HD), distance from center, and relative volume difference.

**Results:**

Prostate gland segmentation showed minimal inter‐observer variability and high agreement with the reference, with a mean DSC of 0.89±0.02 for ROs and 0.90±0.03 for AIRC. In contrast, GTV segmentation demonstrated substantial variability and a high number of false positives and missed lesions when ROs lacked access to the radiology report. PPV significantly improved during the second attempt with access to the radiology report (p=0.03), and similarity metrics improved significantly across all measures (p<0.01). Sensitivity also increased in the second attempt, although not significantly (p=0.0625). AIRC performance was comparable to the ROs' first attempt.

**Conclusions:**

High inter‐observer variability in GTV segmentation was observed when ROs lacked access to the radiology report, underscoring the difficulty of mpMRI interpretation. Providing radiology reports and establishing standardized guidelines for mpMRI interpretation may improve segmentation accuracy and consistency. An Automated segmentation tool could facilitate the first step in the planning workflow by assisting ROs in target definition.

## 1 INTRODUCTION

Prostate cancer (PCa) is the fourth most commonly diagnosed cancer worldwide, with over 1.4 million new cases annually, leading to more than 300,000 deaths each year.^[^ ^1^ ^]^ Surgery and external beam radiotherapy (EBRT) have become the standard of care for men suffering from significant disease.^[^ ^2^, ^3^ ^]^ Recently, studies have shown that escalating radiation dose to the gross tumor volume (GTV) results in an improvement in biochemical disease‐free survival (bDFS) rate compared to conventional treatment, as demonstrated by the FLAME trial.^[^ ^4^ ^]^ As defined by ICRU Report 83, the GTV indicates the gross demonstrable extent and location of the tumor.^[^ ^5^ ^]^ Additionally, focal boosting of GTVs, either with EBRT or brachytherapy (BT), has been associated with improved clinical outcomes and no significant increase in toxicity compared to whole‐gland dose escalation.^[^ ^6^, ^7^, ^8^, ^9^ ^]^

Focal boosting to GTV requires robust segmentation of the visible tumoral lesion during treatment planning. To achieve this, magnetic resonance imaging (MRI) is used for its superior soft‐tissue contrast, over other imaging modalities. Multiparametric MRI (mpMRI) can facilitate the identification of PCa compared to T2w sequence alone.^[^ ^10^, ^11^, ^12^ ^]^ mpMRI exam consists of a combination of a T2 weighted (T2w) sequence, apparent diffusion coefficient (ADC) map derived from the diffusion weighted imaging (DWI) data, in addition to a dynamic contrast enhanced (DCE) T1 weighted (T1w) sequence. However, identifying GTV on mpMRI can be challenging for radiation oncologists (ROs) who are not specifically trained in prostate mpMRI interpretation. Lui et al.^[^ ^13^ ^]^ studied RO's accuracy in contouring GTV on mpMRI and 44 participants completely missed the target in 18% of attempts, illustrating the difficulty for ROs to delineate tumors accurately on mpMRI sequences.

Multidisciplinary collaboration between radiologists and ROs has been shown to improve lesion identification with histological confirmation; such collaboration or access to pre‐treatment mpMRI with diagnostic interpretation is not consistently available and may vary by center.^[^ ^14^, ^15^, ^16^, ^17^ ^]^ In this context, ROs delineation of the GTV on mpMRI in absence of support from radiologists is not well known.

As a potential replacement for radiology report, recent studies have explored the use of artificial intelligence (AI), specifically deep learning (DL), to automatically delineate GTV on mpMRI.^[^ ^18^ ^]^ A major challenge remains, as the deployment of such algorithms and their performance can be affected by variations in mpMRI acquisition protocols.^[^ ^19^ ^]^

In a study by Zhong et al., one of the principal barriers identified by ROs for the implementation of focal boost to the lesion in a clinical setting was the challenge associated with access to high‐quality MR images and the lack of training to identify GTV(s) on mpMRI images.^[^ ^20^ ^]^ The goal of this study is to assess the performance of a commercial AI‐based segmentation software to explore its potential role in supporting consistent delineation during radiotherapy planning. Considering the variability in radiation oncologist mpMRI training and access to radiology reports, we compare the segmentation performance of the prostatic gland and GTVs by ROs with and without radiology input to the AI‐based segmentation. To our knowledge, this is the first work to investigate whether a commercial AI‐based segmentation software can segment prostate and GTV on mpMRI with an accuracy comparable to radiologists and ROs with and without a radiology report.

## 2 MATERIALS and METHODS

### 2.1 Patient population

This retrospective cohort included 20 patients with histologically confirmed prostate adenocarcinoma. Nine out of twenty patients (45%) underwent systematic biopsies, in which the prostate was sampled indiscriminately, while the remaining eleven (55%) underwent combined systematic and MRI‐targeted biopsies. All patients were treated at our facility and had not received any prior treatment. Institutional review board approval was obtained. Low to intermediate risk patients were eligible according to the following criteria: Clinical stage T1‐T2, Nx or N0, Mx or M0, PSA ≤ 20 ng/mL, Gleason 6–10. Patients must have undergone mpMRI at our facility prior to treatment delivery. Detailed patient characteristics are displayed in Table 1.

**TABLE 1:** Patients characteristics of the patients that participated in the study.

Characteristics of patients	Number of patients = 20
**Followed hormone therapy?**	
Yes	9/20 (45%)
No	11/20 (55%)
**Age** (median (range))	70 (55–83)
**Gleason score**	
6	2/20 (10%)
3+4	8/20 (40%)
4+3	8/20 (40%)
810	2/20 (10%)
**PI‐RADS score**	
PI‐RADS 3	2/20 (10%)
PI‐RADS 4	8/20 (40%)
PI‐RADS 5	10/20 (50%)
**Lesion characteristics**	
Mean volume (cc (range))	1.01 (0.11‐3.74)
Total number of lesions	*N*=26
Mean number of lesions per patient (range)	1.3 (1–3)
**Biopsy**	
Systematic	9/20 (45%)
Systematic + MRI guided	11/20 (55%)

### 2.2 Imaging protocol

mpMRI was performed following the Prostate Imaging Reporting and Data System (PI‐RADS V2.1) guidelines,^[^ ^21^ ^]^ and a board‐certified radiologist's report was obtained for each case. Imaging was performed using a MAGNETOM Sola 1.5 T scanner (Siemens Healthineers, Erlangen, Germany). Image acquisition was performed using the spine and the Body 18 channels coil. No endorectal coil was used, as per clinical guidelines at our center. The mpMRI imaging protocol consisted of a 2D and 3D T2w Turbo Spin Echo (TSE), DWI using a readout‐segmented echo‐planar imaging technique (RESOLVE) to reduce geometric distortion and a T1w with Dynamic Contrast Enhancement (DCE) using GRASP‐VIBE technique. This DCE sequence was performed before, during and after a bolus injection of contrast agent (*Gadovist*) with a temporal resolution of 5 s. Detailed scanning parameters are supplied in Table 4 of the supporting material.

### 2.3 Prostate gland and GTV segmentation

In total, six board‐certified ROs from our center, consisting of one fellow, and 5 ROs were asked to delineate the prostate gland and GTV(s) corresponding to the visible PCa lesion(s) identified on mpMRI. Before this, they received training from a radiologist on the PI‐RADS score system, the usefulness of the different MR weighting as well as PCa lesion appearance on mpMRI. They were asked to perform the segmentation two times and were blind each time to their peer's contours. For their first attempt, ROs only had access to the biopsy report, indicating the Gleason Score in addition to the radiology PI‐RADS score. A minimum of 8 months elapsed before their following attempt. For the second attempt, ROs were given the full written radiology report, indicating the location of GTVs on mpMRI, including an arrow on one slice of the T2w volume. ROs delineated GTV on the 3D T2w TSE sequence as primary, with access to the other sequences as secondary. They were instructed to perform delineation using the provided sequence without any patient‐specific guidance, adhering to their usual clinical workflow. All contours were done in the radiotherapy treatment planning system (TPS) used at our clinic (RayStation 10B, Raysearch Lab, Sweden).

Additionally, an automatic cloud‐based segmentation tool, using AI was employed on the same dataset to assess its performance relative to that of ROs. AI‐Rad companion (AIRC) MR Prostate (version VA60, Siemens Healthineers, Erlangen, Germany) was designed for whole prostate segmentation, detection of suspected lesions and classifications based on PI‐RADS for PCa diagnostic assistance.^[^ ^22^ ^]^ Therefore, it can automatically delineate the whole prostatic gland, as well as suspicious cancerous lesions. This product was not initially intended to be used in the radiation oncology setting and is currently not clinically approved for that usage in North America. AI segmentation of the prostate gland and GTV was performed on the same dataset, as available for the ROs and used for comparison.

### 2.4 Reference segmentation

Two board‐certified radiologists, each with a fellowship in abdominal MRI and subspecialty training in prostate imaging independently delineated the same dataset. The radiologists had access to the prior biopsy results for each patient, including Gleason score and the location of positive cores, which informed their GTV segmentations. A consensus segmentation was obtained using the STAPLE algorithm,^[^ ^23^, ^24^ ^]^ which generates a reference contour representing the average segmentation of each structure based on the inputs from both experts. To obtain the STAPLE segmentation for the prostate gland and GTV, we used the implementation of the algorithm available in the SimpleITK (v2.4.1) library, using Python (v3.13.2). For 5 patients included in the study, the radiologists did not reach an agreement (Dice = 0). For these cases, the radiologists delineated a contour based on consensus, after consulting with each other. Agreement between the two experts was assessed, and results are displayed in Figure 13 in the supporting document V

### 2.5 Analysis

Radiotherapy Structure Sets in DICOM‐RT format were exported from the TPS to obtain similarity metrics. Dice similarity coefficient (DSC), Hausdorff distances (HD), distance from center and relative volume variation were obtained using 3DSlicer (v5.2.2) Radiotherapy Module to compare the ROs segmentations and AIRC to the reference (i.e., the STAPLE or consensus segmentation).^[^ ^25^, ^26^ ^]^ In addition, an identification of lesion analysis was performed to obtain the sensitivity ($\text{TP}/\left[\text{TP}+\text{FN}\right]$, where TP: True positive, FN: False negative) and PPV (PPV=TP/[TP+FP], where FP: False positive) for both segmentation approach. GTV with DSC ≥ 0.1 compared to the reference was considered as a TP, whereas lesions with insufficient overlap to the reference (DSC < 0.1) were labelled as FP. Any additional lesion segmented by ROs or AIRC which did not overlap with any reference lesion was labelled as an FP, also.^[^ ^27^ ^]^ Statistical analysis was performed in Python (v3.13.2) using SciPy.stats module (v1.15.2). Shapiro–Wilk's test was performed to assess normality of the data distribution, and a Wilcoxon Signed‐Rank test with a significance level set at α=0.05 was used.

## 3 RESULTS

A total of 240 segmentation sets were obtained from the group of ROs, and 20 sets for the automated segmentation tool. Across both attempts made by the ROs, a total of 307 GTVs coming from the 240 segmentations sets were compared to the reference GTVs. In the first attempt by the ROs, 4 cases were not delineated by one of the physicians (RO4) and were therefore excluded from the pairwise analysis in the second attempt.

### 3.1 Identification of lesion performance

The performance for lesion identification is presented in Table 2. Without access to the radiology report, the ROs correctly identified 101 true lesions but also generated 50 FP and missed 50 lesions, which were considered FN, yielding a total of 151 predicted lesions. As for AIRC, it identified correctly 16 lesions out of the 26 reference GTVs, with 4 FP and 10 FN. In this first attempt, the ROs achieved a sensitivity of 66.9%, which was comparable to AIRC at 61.5%, with no statistically significant difference between the two (p=0.44). However, AIRC demonstrated a significantly higher PPV (80.0% vs. 66.9%, p=0.03), as shown in Table 2.

**TABLE 2:** Sensitivity and positive predictive value (PPV) for segmentation attempt without and with support of the radiology report, as well as results for the automated segmentation tool.

Group	True positive rate (%)	False positive rate (%)	False negative rate (%)	Sensitivity (%)	Positive predictive value (%)
Radiation oncologists (without radiology report)	66.9	33.1	33.1	66.9	66.9
Radiation oncologists (with radiology report)	86.5	10.6	13.5	86.5	89.4
AIRC	61.5	20.0	38.5	61.5	80.0

When the ROs had access to the radiology report, both sensitivity and PPV improved (p=0.063 and p=0.031 respectively), indicating better agreement with the reference lesions defined by the STAPLE contours. In this second attempt, the ROs outperformed AIRC, achieving a significantly higher sensitivity (p=0.03) and a higher, although not statistically significant PPV (p=0.13).

### 3.2 Similarity metrics analysis

**FIGURE 1:**
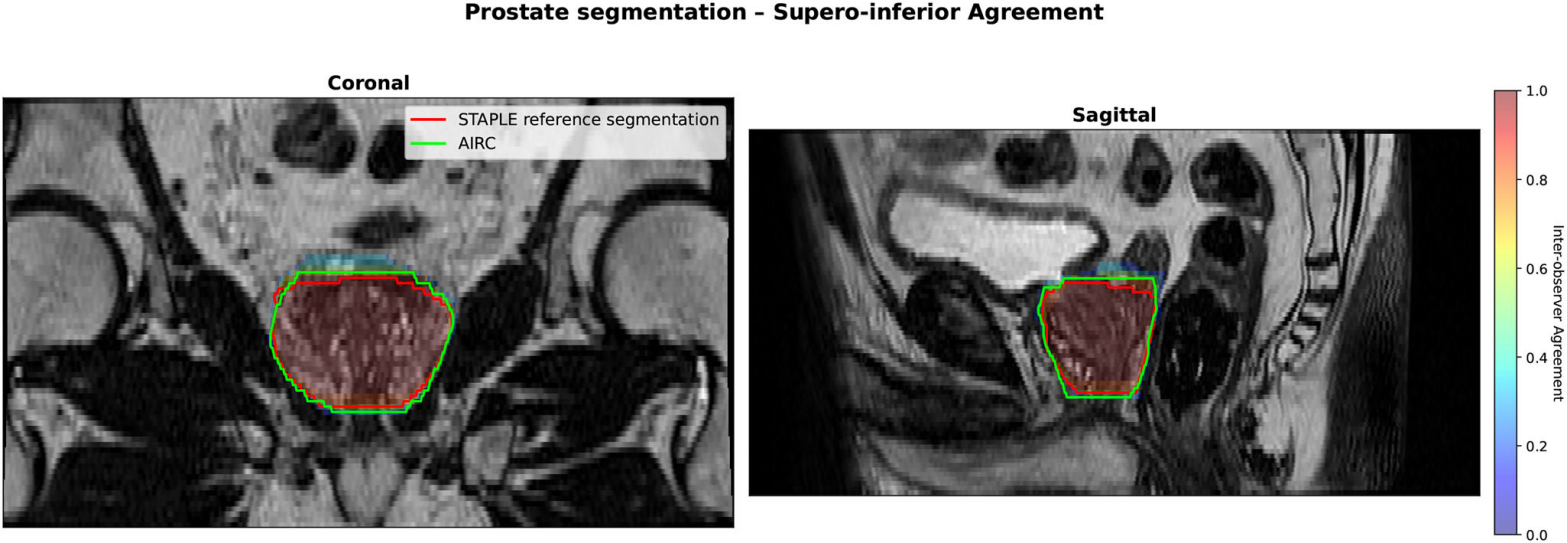
Comparison of the segmentation for the prostate by the RO group (heatmap) with the STAPLE reference segmentation (red) and AIRC (green).

Table 3 shows the similarity metrics obtained for ROs and AIRC for both prostate and GTV. After analysis of the first attempt, ROs were not asked to re‐segment the prostate gland as results obtained were deemed good enough compared to the STAPLE prostate contour. AIRC prostate contour resulted in a slightly higher, but significant (p=0.02) DSC compared to ROs. For the other metrics, AIRC resulted in a smaller HD and distance to center where only HD95%, HD Average were significantly lower (p<0.05). Main difference in contours of the prostate can be seen at the base and apex, as illustrated in Figure 1. Similarity metrics figures for the prostate are supplied in Figures 4–7 in the supporting document V

**TABLE 3:** Results obtained for the different similarity metrics (Dice similarity coefficient (DSC), Hausdorff distances (HD), distance to center and relative volume difference) analyzed, compared to both attempt by the ROs and the automatic segmentation tool (AIRC).

Structure	Group	DSC (0–1)	HD 95% (mm)	HD average (mm)	HD max (mm)	Distance to center (mm)	Relative volume difference (%)
Prostate	Radiation oncologists (without radiology report)	0.89±0.02	3.84±0.55	1.37±0.37	6.16±1.80	1.37±0.81	16.67±10.33
	AIRC	0.90±0.03	3.28±1.00	1.19±0.33	5.56±1.60	1.18±0.74	3.29±7.73
GTV	Radiation oncologists (without radiology report)	0.36±0.19	6.33±3.43	3.47±4.56	8.44±7.33	5.67±6.25	182.37±262.68
	Radiation oncologists (with radiology report)	0.49±0.14	4.36±1.92	2.23±3.21	6.50±4.86	3.48±4.26	102.58±145.40
	AIRC	0.34±0.29	4.68±6.09	2.56±4.66	6.38±7.53	5.43±6.88	18.27±68.49

**FIGURE 2:**
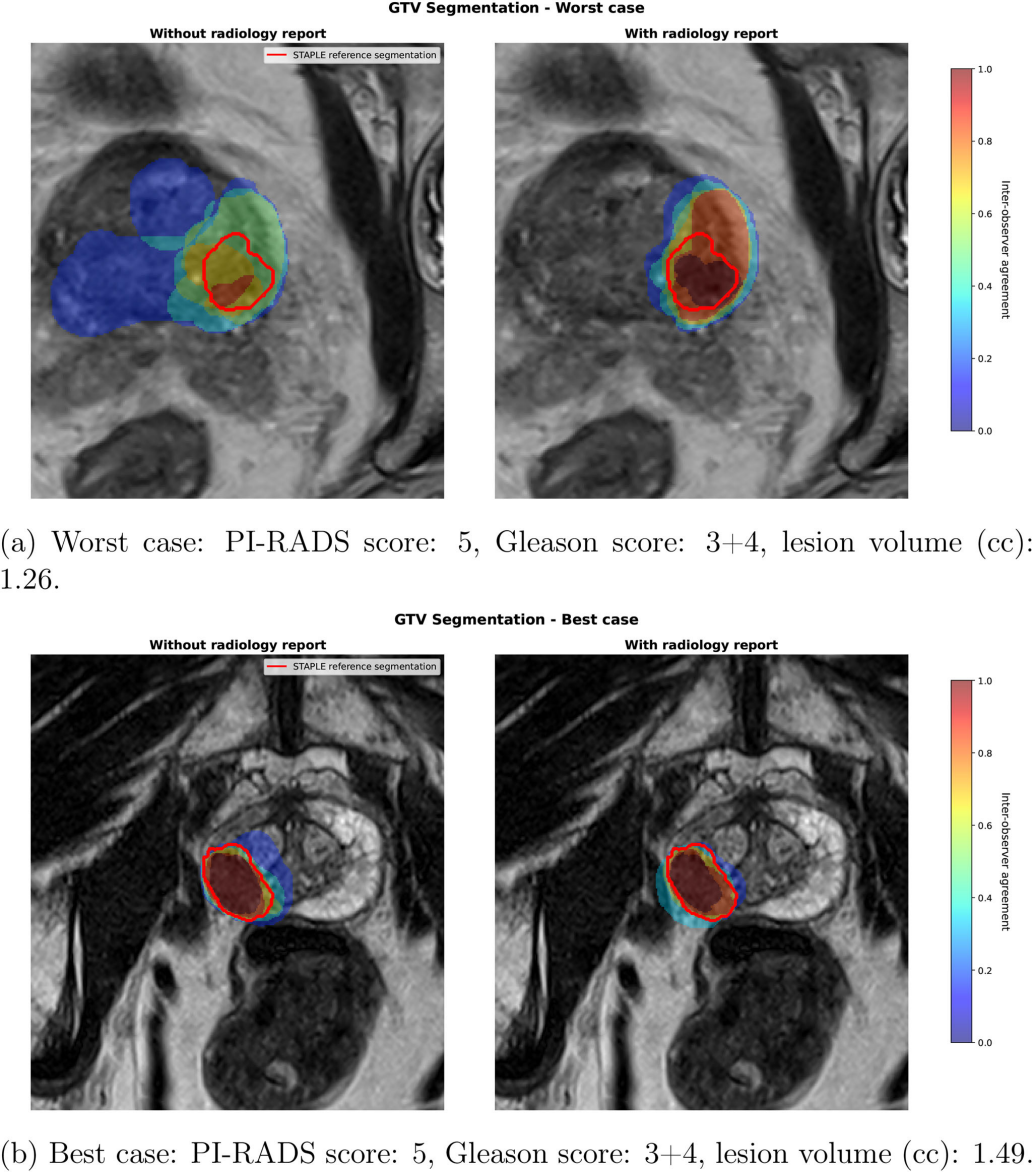
Effect of the addition of the radiology report on inter‐observer agreement between radiation oncologists (ROs) for the GTV contour. Comparison between the worst and best case in the dataset. STAPLE reference GTV shown in Red.

As for the GTV contours, a greater inter‐observer variability was seen when ROs did not have access to the radiology report. DSC is greatly reduced compared to the prostate (0.36±0.19 vs. 0.89±0.02), indicating a small overlap between ROs defined GTV and STAPLE reference GTV. A similar trend is observed as well with the other metrics, showing higher HD metrics and distance to center. The effect of adding the radiology report can be seen in Figure 2.

**FIGURE 3:**
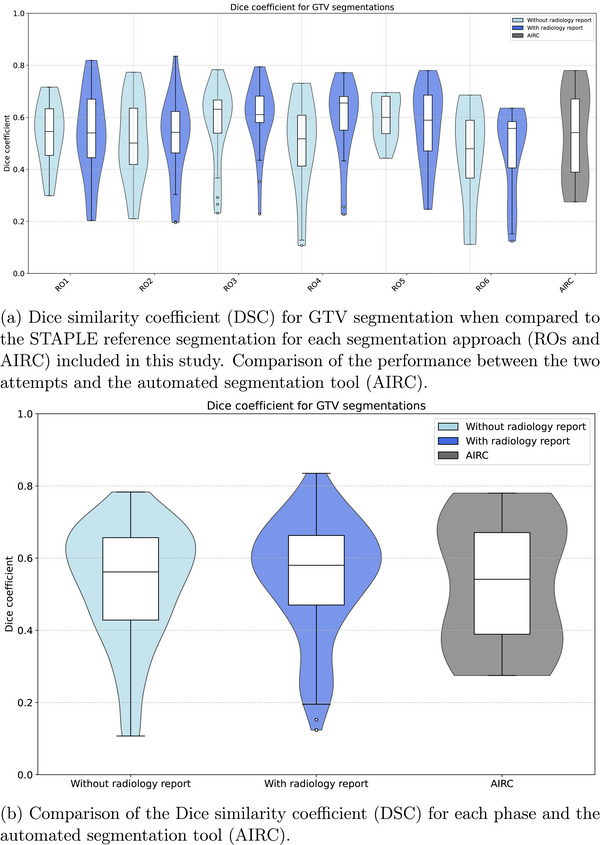
Dice similarity coefficient (DSC) obtained after comparison to the STAPLE reference segmentation across both phases illustrated by each segmentation approach. Comparison resulting in a DSC < 0.1 were excluded.

Figure 3a shows the improvement in overlap by the DSC between both attempts by ROs. For each physician, the attempt resulting in a higher DSC was the one performed with the radiology report, with a mean improvement of 37.3% (range: 11.6%–83.3%). However, a statistically significant improvement was observed only for RO1, RO5, and RO6 (p<0.05), who also had the lowest initial performance scores. When results from all ROs are combined for each phase, as illustrated in Figure 3b, DSC was similar across all attempts, without a significant difference between the groups (p>0.05). A similar trend is observed for the HD95% metric, as seen in Figure 8 in supplementary data. Each RO had a lower HD95% compared to the reference GTV in their second attempt, but only RO2 and RO5 significantly improved their score (p<0.05). The mean HD and HD max were also significantly reduced (p<0.001) by 41.0% and 27.8%, respectively, for all physicians in their second attempt. A comparable improvement for distance to center and relative volume difference was observed. The corresponding results are illustrated in Figures 8–12 (Supporting Document V).

## 4 DISCUSSION

The objective of this study was to evaluate the performance of a commercial AI‐based segmentation software and explore its potential role in supporting consistent delineation during radiotherapy planning. Given the variability in ROs mpMRI interpretation training and their access to the radiology report, we compared the segmentation performance of the prostate gland and GTVs by ROs with and without access to radiology input to the AI‐based segmentation.

The prostate segmentation performance achieved by the ROs was consistent with previously published results.^[^ ^28^, ^29^ ^]^ Similarly, the automated segmentation tool (AIRC) demonstrated a high level of agreement with the reference contour, yielding a mean DSC of 0.90±0.03, which aligns with findings from earlier studies.^[^ ^19^, ^30^ ^]^ As reported in the literature, the primary discrepancies in segmentation for both ROs and AIRC were observed at the supero‐inferior extremities of the prostate gland, particularly at the base and apex.^[^ ^29^ ^]^ While automated segmentation can significantly reduce the time required for treatment planning, careful validation of the generated contours remains essential to ensure accurate and clinically acceptable delineations.

As for the detection and delineation of GTVs, our study demonstrates that it is challenging for ROs to correctly identify clinically significant cancer on mpMRI exams when no input from radiology is provided. Inaccurate or omitted focal boosting of the dominant lesion may reduce treatment efficacy and increase the likelihood of recurrence, given that failures typically occur at the site of the primary tumor.^[^ ^31^ ^]^ Although tumor delineation accuracy and precision had already been investigated in the past, none of the current studies highlighted the influence of having a radiologist review the segmentation of the GTV. No definitive guidelines exist on whether radiology input should be systematically involved in evaluating GTV segmentations. Our study demonstrates that having access to the radiology report significantly increases segmentation quality. This correlates to a recent meta‐analysis showing that radiologist involvement in peer review led to plan modification in nearly half of cases (49.4%) compared with only 25.0% when radiologists were not involved.^[^ ^15^ ^]^ A potential explanation for the variability and erroneous segmentation of GTV is the inadequate use of the different available MRI sequences (anatomical and functional weightings) as previously highlighted in a related study.^[^ ^32^ ^]^ This is further reflected in a study assessing radiology training during radiation oncology residency, where a majority of residents felt their radiology training was suboptimal.^[^ ^33^ ^]^ Standardizing the workflow among physicians and establishing clear guidelines on the use of mpMRI for GTV delineation, similar to those available for whole‐prostate segmentation, could improve the accuracy of GTV identification on mpMRI.

Automated detection and segmentation of clinically significant PCa using DL has been explored in recent studies, with varying degrees of success.^[^ ^34^, ^35^, ^36^ ^]^ Yet, no commercial solutions have been evaluated for target delineation. The results of the present study indicate that the ROs outperformed the automated segmentation tool when provided with access to the radiology report. Importantly, the evaluated AI‐based tool was originally designed for diagnostic use following mpMRI to support lesion detection and biopsy decision between radiologist and urologist. In contrast, radiotherapy treatment planning represents a distinct clinical workflow with higher accuracy requirements and direct implications on dose delivery. Although such tools hold promises for the future, careful implementation is essential. Off‐the‐shelf solutions, such as the one evaluated in this study, may have limited applicability depending on the characteristics of the training data, including sequence weighting and parameters, MRI field strength, and patient population. AIRC was mainly trained on radiology patients at a field strength of 3T (90% of 3000 cases), which could explain the results obtained in this study for radiotherapy with a 1.5T magnet. AIRC could nonetheless be used as an initial step in the segmentation workflow allowing ROs to review, refine, or reject the proposed lesion contours as needed to reduce the number of complete misses. Training the algorithm explicitly from radiotherapy data may also improve its precision for treatment planning purposes.

This study has several limitations. First, it involved a single group of ROs and radiologists from the same institution, which may limit the generalizability of the findings. Second, the study was conducted on a relatively small cohort of patients. Although the majority of participants (≈90%) had clinically significant PCa on mpMRI, as assessed by a radiologist, the number of FP and FN was higher than expected. Also, no pathological validation of the reference segmentation was performed, as no patient included in this study underwent radical prostatectomy. Additional studies involving larger, multi‐institutional cohorts are needed to standardize GTV segmentation procedures, particularly in the context of focal boost treatment strategies, such as the one proposed in the FLAME trial.

## 5 SUMMARY AND CONCLUSIONS

In conclusion, this study highlights substantial inter‐observer variability in GTV delineation on mpMRI when ROs do not have access to the radiology report. Prostate segmentation, in contrast, showed high agreement across observers and with the AI auto‐contouring software. A DL‐based automated segmentation tool demonstrated potential to support the delineation process when lacking radiology input and reduce associated workload. However, standardized guidelines for the use of mpMRI are essential to ensure accurate identification of clinically significant PCa and to fully leverage the therapeutic benefits of focal boost radiation therapy.

## CONFLICTS OF INTEREST STATEMENT

P.D. receives a scholarship from the Ministry of Health and Social Services (MSSS) of the Province of Quebec.

A.‐G.M. receives funding from Varian, a Siemens Healthineers company, for conducting this study.

L.A. is funded in part through the Natural Sciences and Engineering Research Council of Canada (NSERC) Discovery Grant #2024‐04198.

E.P. receives funding from Varian, a Siemens Healthineers company, and Siemens Healthineers for conducting this study.

All other authors declare no conflict of interest.

## FUNDING INFORMATION

Varian, a Siemens Healthineers Company, provided financial support for the conduct of this study.

## Supporting information

Supporting Information

## Data Availability

Data generated or analyzed during the study are not available for sharing.
