# Supplementary material for: Assessing inter‐observer variability in prostate and GTV segmentation on mpMRI: A comparison between radiation oncologists and AI‐based method
Source: J Appl Clin Med Phys. 2026 Apr 3;27(4):e70563. doi: 10.1002/acm2.70563 (PMC13052058; doi:10.1002/acm2.70563)
Supplement: Supplementary file 1 — Supporting Information [file ACM2-27-e70563-s001.pdf]

Appendix

Scanning parameters

Table 4: Scanning protocol and parameters for the patients scanned in this study.

Sequence	FoV (mm)	Matrix size (RO x Phase)	TE/TR/Turbo Factor (ms/ms/-)	Slice thickness (mm)
T2w 3D SPACE	240 x 205	352 x 285	99/1600/100	1.5
T2w TSE	200 x 200	336 x 289	83/5120/25	3.0
T1w DCE	240 x 240	224 x 224	2.35/4.23/1	3.5
RESOLVE DWI	200 x 200	116 x 116	77.18/4170/-	3.0

Supporting Material

Prostate similarity metrics

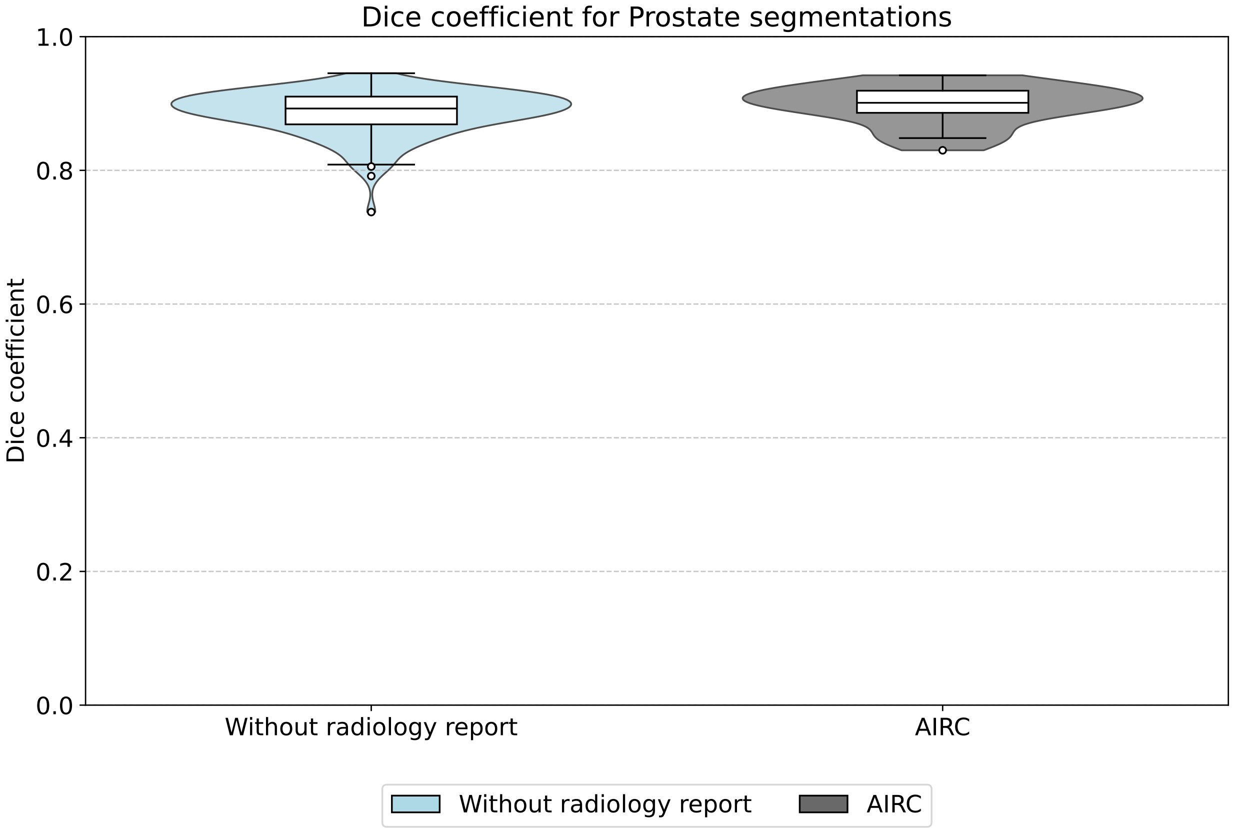

Figure 4: Comparison of the Dice similarity coefficient (DSC) obtained for the group of radiation oncologists (ROs) vs the automated segmentation tool (AIRC).

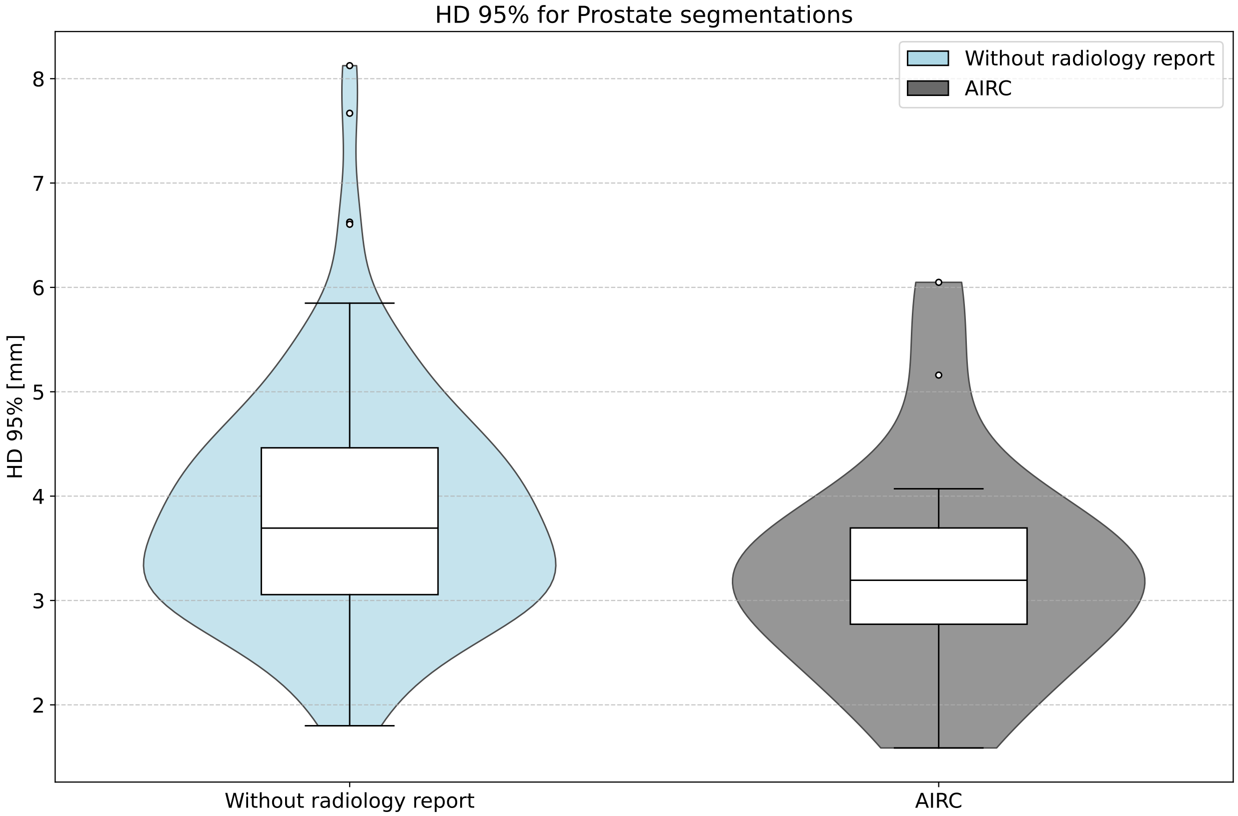

Figure 5: Comparison of the Hausdorff Distance in the 95% CI (HD95%) obtained for the group of radiation oncologists (ROs) vs the automated segmentation tool (AIRC).

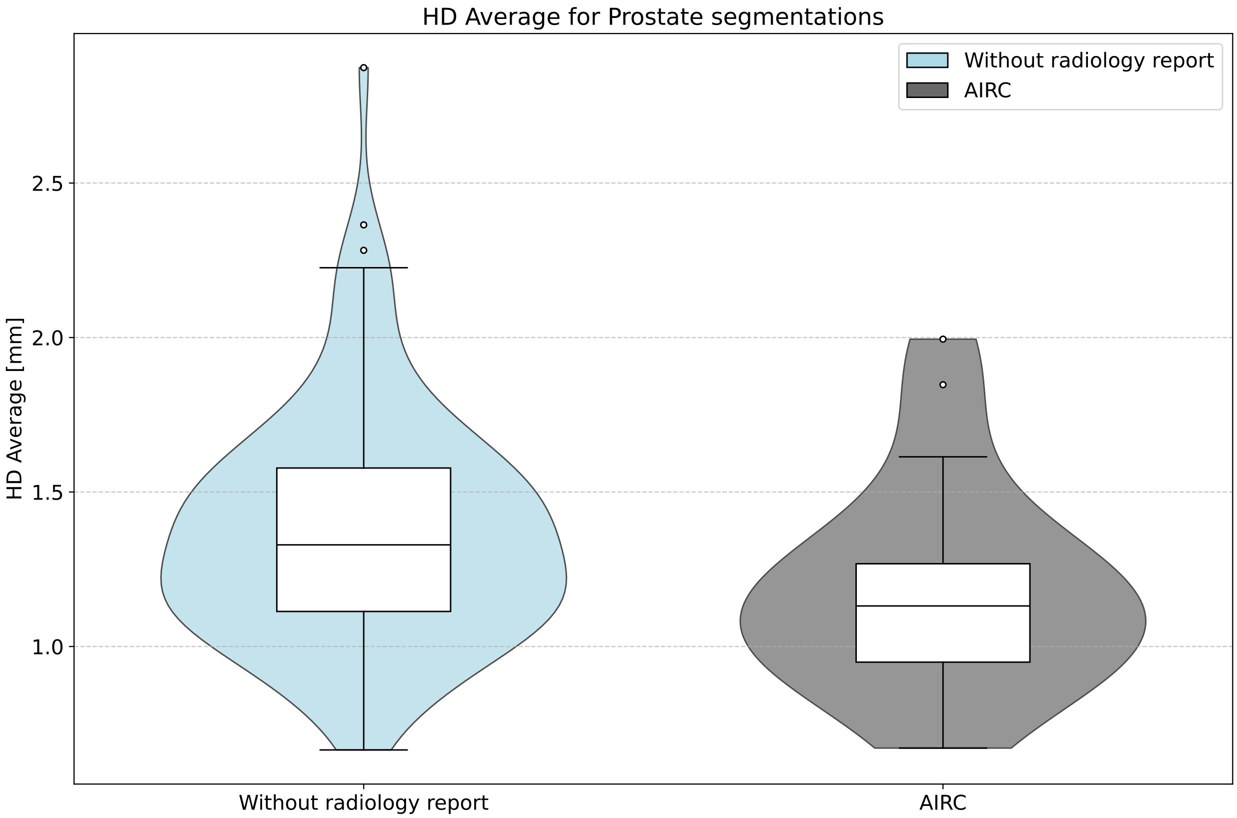

Figure 6: Comparison of the average Hausdorff Distance (HDmean) obtained for the group of Radiation Oncologists (ROs) vs the automated segmentation tool (AIRC).

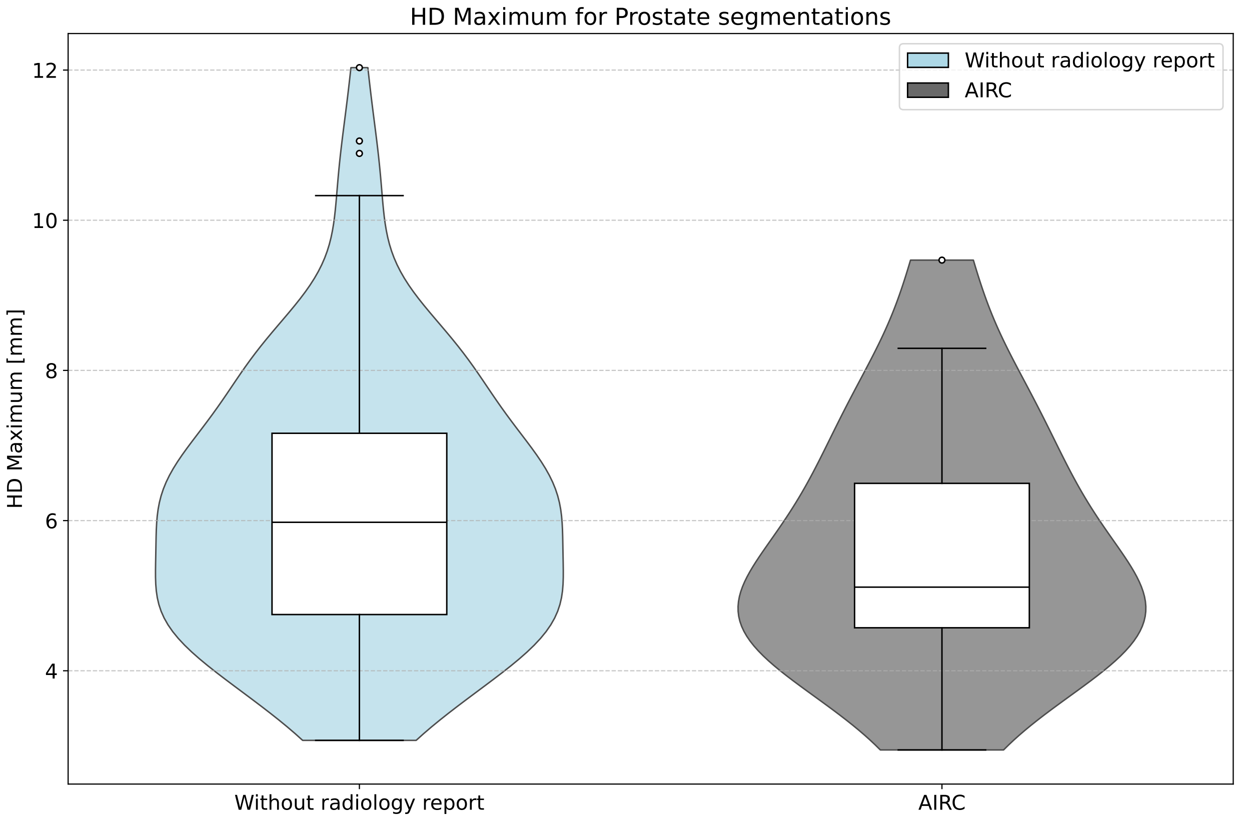

Figure 7: Comparison of the maximum Hausdorff Distance (HDmax) obtained for the group of Radiation Oncologists (ROs) vs the automated segmentation tool (AIRC).

GTV similarity metrics

V. SUMMARY AND CONCLUSIONS

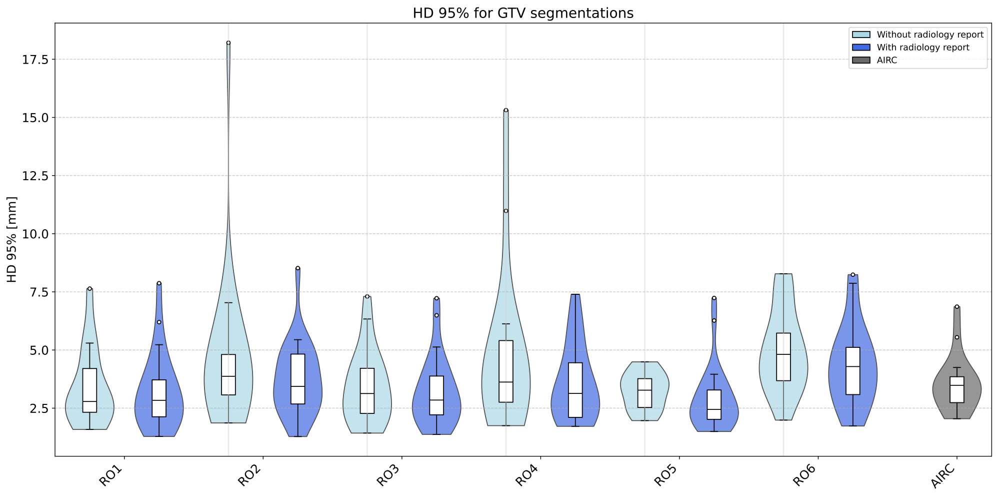

(a) Hausdorff distance in the 95% CI (HD95%) for GTV segmentation when compared to the STAPLE reference contour for each segmentation approach included in this study. Comparison of the performance between the two attempts and the automated segmentation tool (AIRC).

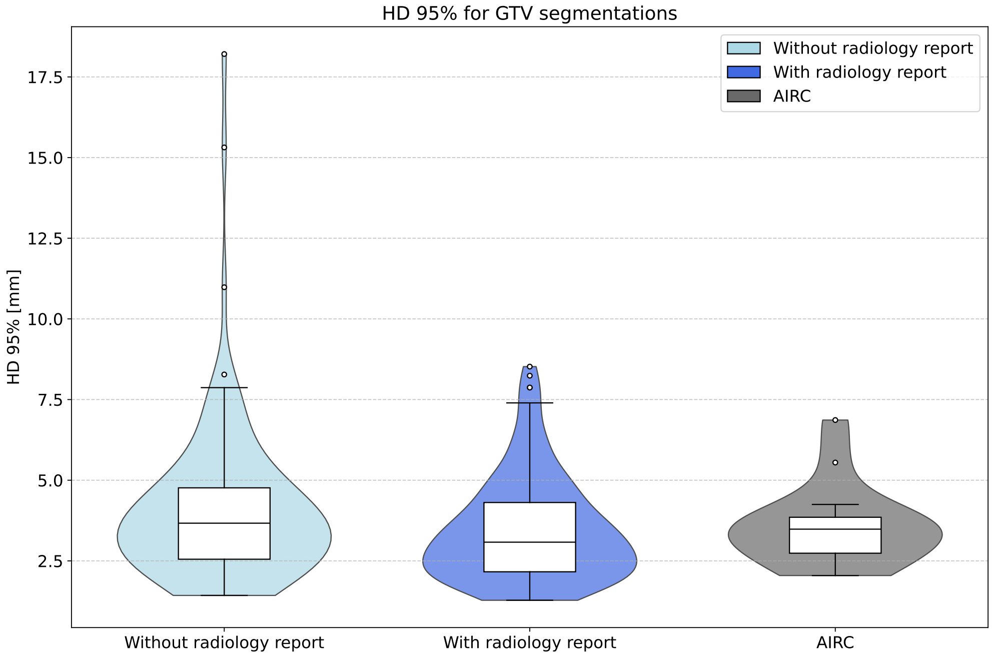

(b) Comparison of the Hausdorff distance in the 95% CI (HD95%) for each phase and the automated segmentation tool (AIRC).

Figure 8: Hausdorff distance in the 95% CI (HD95%) for each ROs and across the two phases.

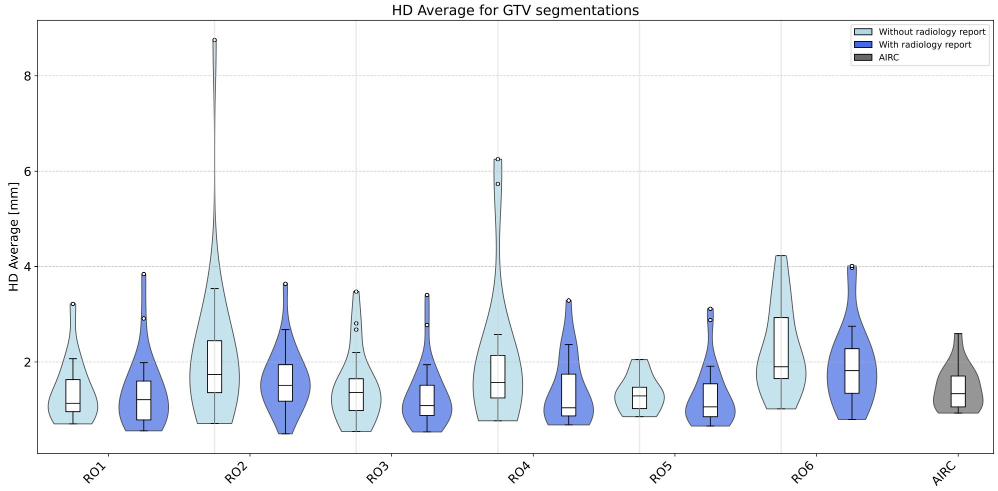

(a) Mean Hausdorff Distance (HD Mean) for GTV segmentation when compared to the STAPLE reference contour for each group included in this study. Comparison of the performance between the two attempts and the automated segmentation tool (AIRC).

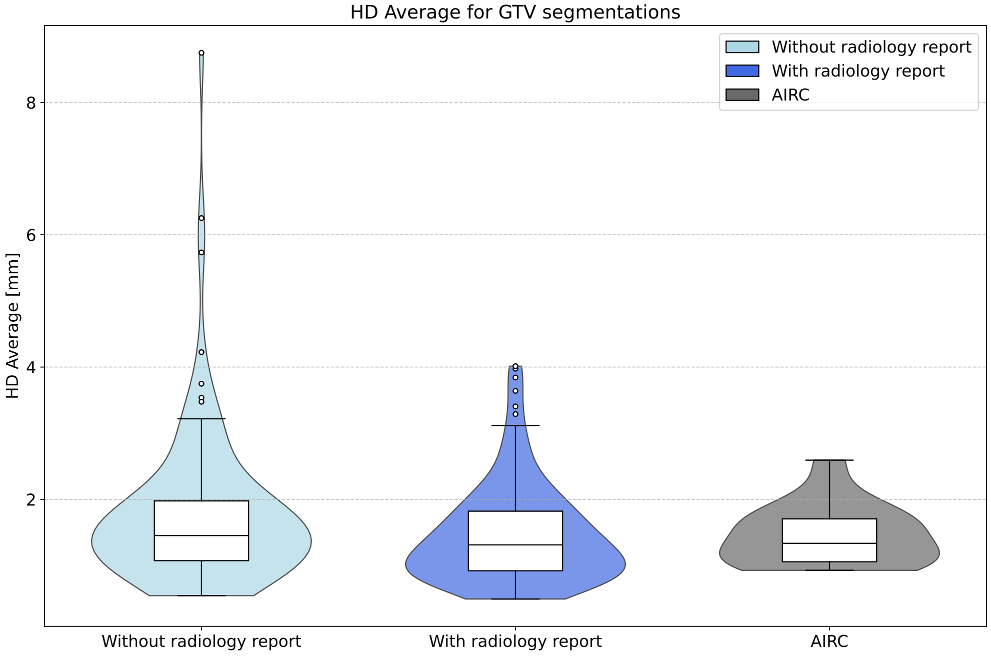

(b) Mean Hausdorff Distance (HD Mean) for each phase and the automated segmentation tool (AIRC).

Figure 9: Mean Hausdorff distance (HD Mean) for each ROs and across the two phases.

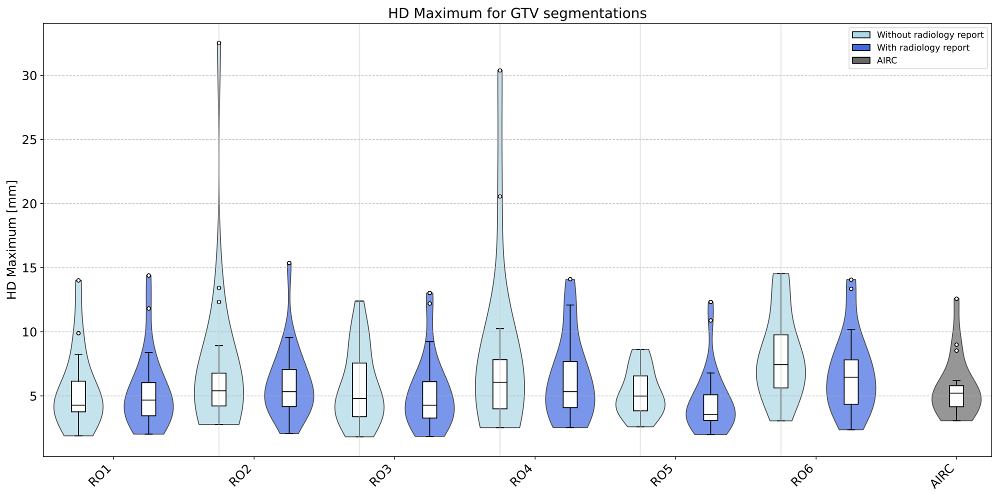

(a) Maximum Hausdorff Distance (HD Max) for GTV segmentation when compared to the STAPLE reference contour for each group included in this study. Comparison of the performance between the two attempts and the automated segmentation tool (AIRC).

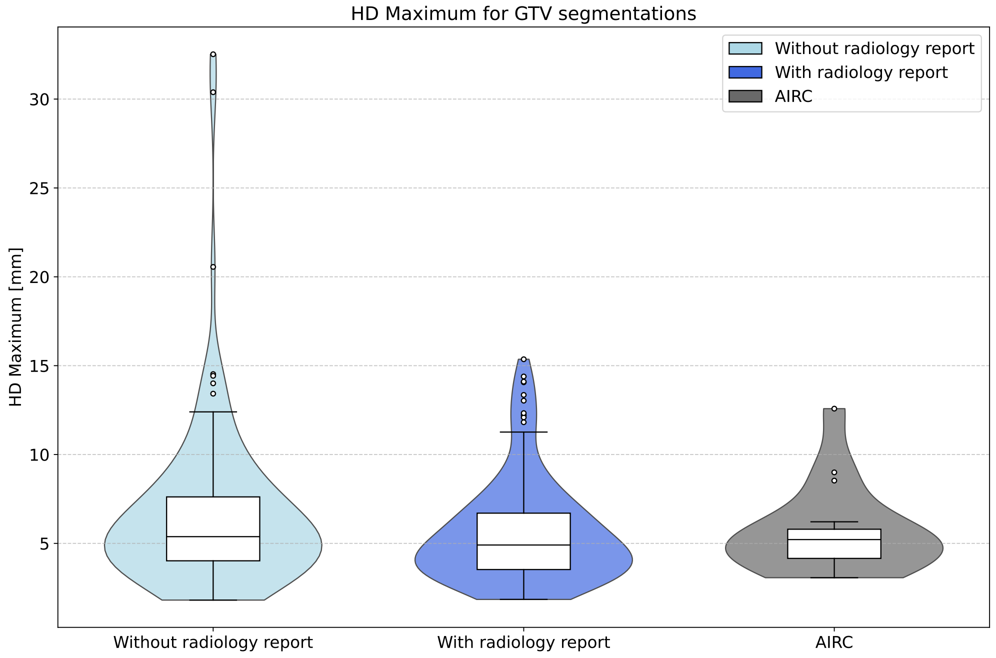

(b) Maximum Hausdorff Distance (HD Max) for each phase and the automated segmentation tool (AIRC).

Figure 10: Maximum Hausdorff Distance (HD Max) for each ROs and across the two phases.

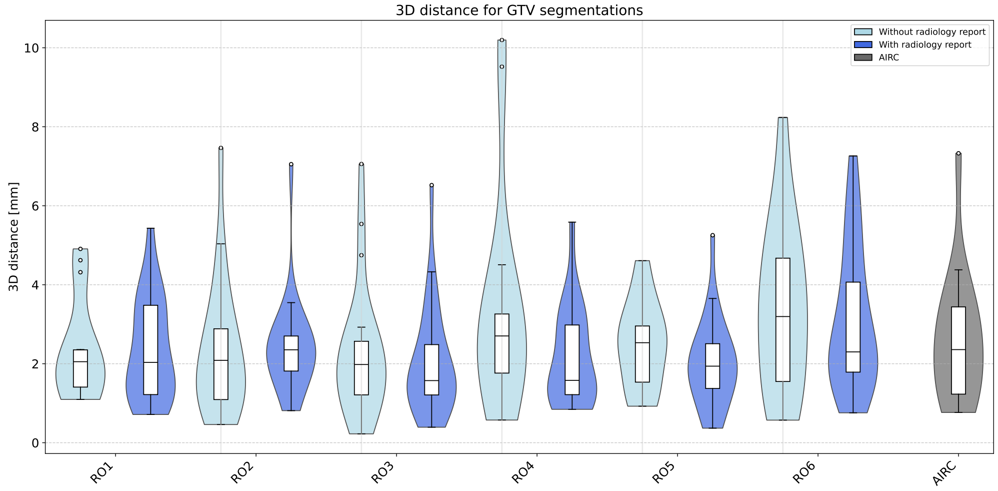

(a) Distance to center (3D distance) for GTV segmentation when compared to the STAPLE reference contour for each group included in this study. Comparison of the performance between the two attempts and the automated segmentation tool (AIRC).

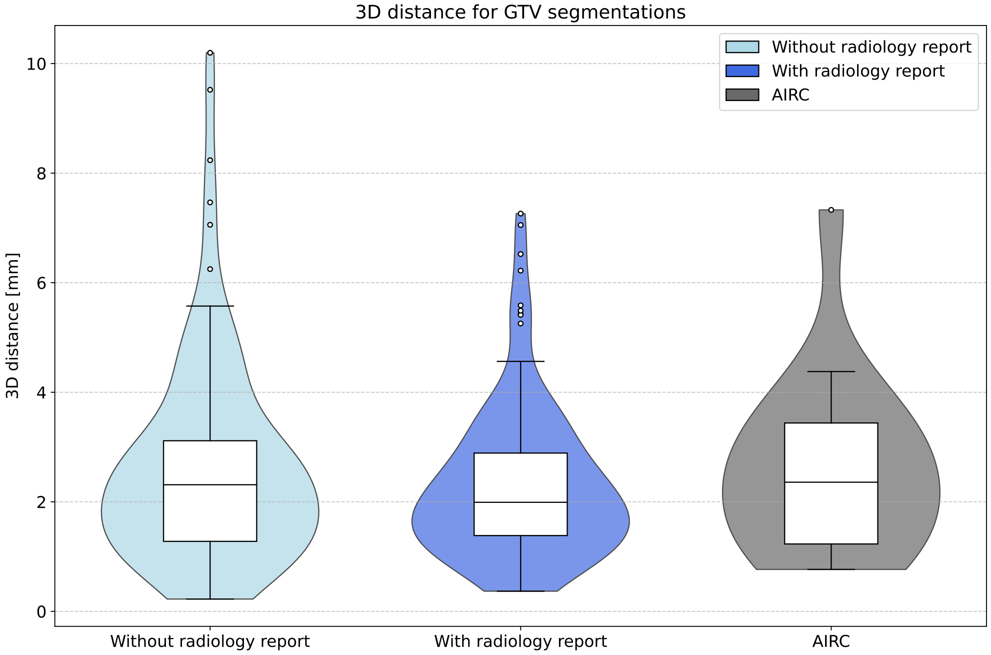

(b) Distance to center (3D distance) for each phase and the automated segmentation tool (AIRC).

Figure 11: Distance to center (3D distance) for each ROs and across the two phases.

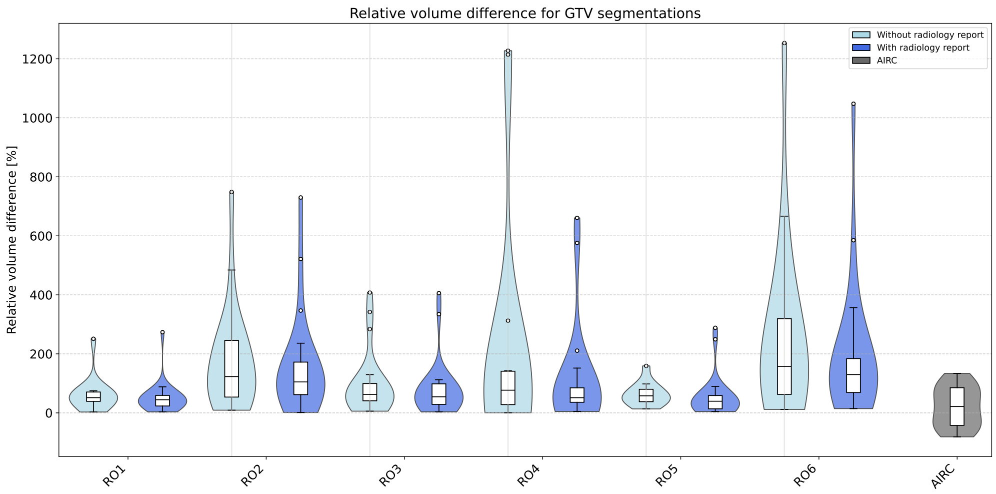

(a) Relative volume difference (%) for GTV segmentation when compared to the STAPLE reference contour for each group included in this study. Comparison of the performance between the two attempts and the automated segmentation tool (AIRC).

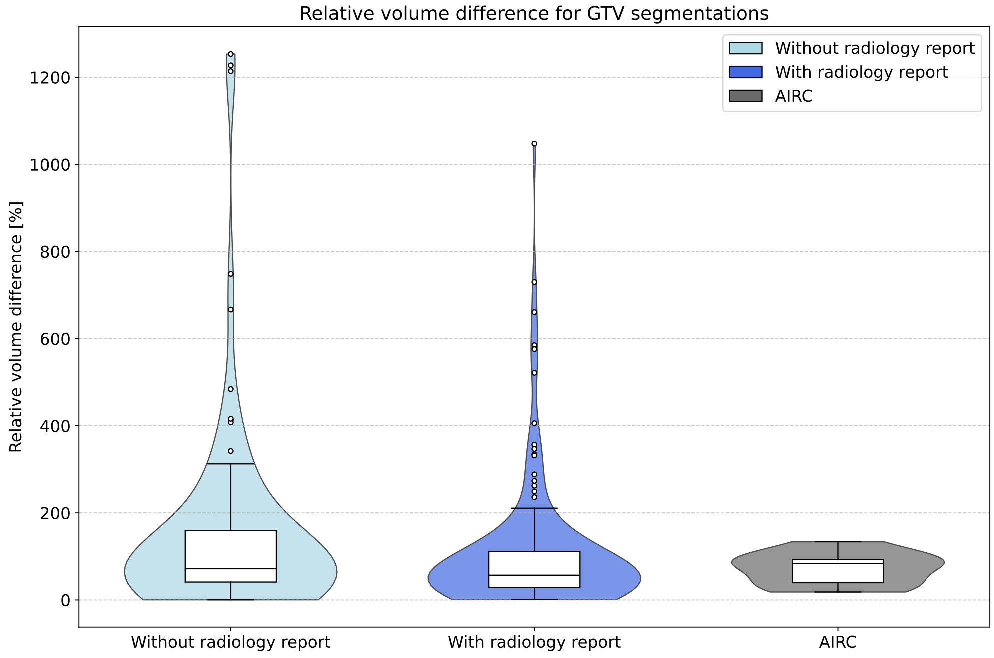

(b) Relative volume difference (%) for each phase and the automated segmentation tool (AIRC).

Figure 12: Relative volume difference (%) for each ROs and across the two phases.

Expert agreement

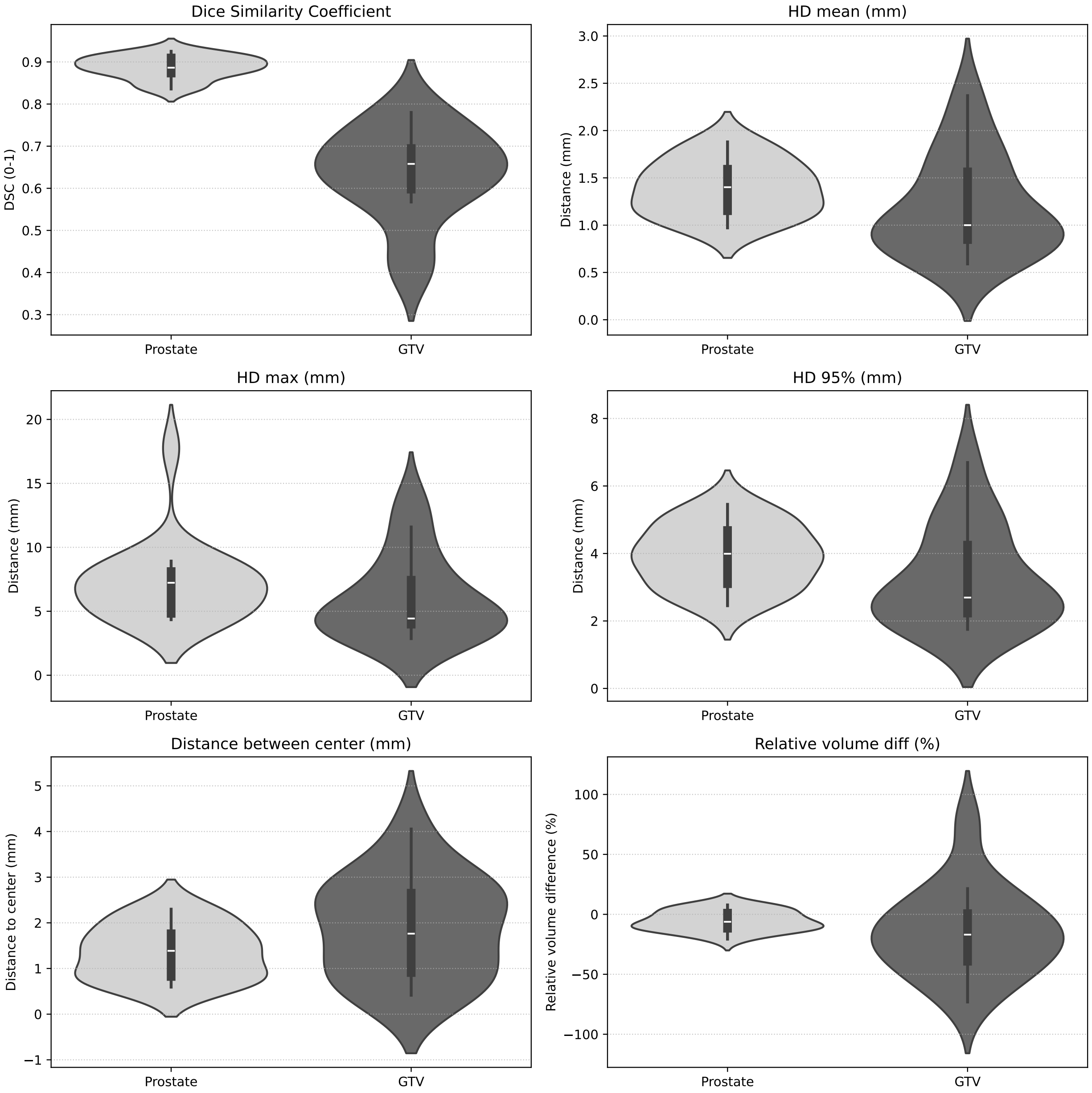

Figure 13: Similarity metrics obtained from the comparison of the two experts for each structure.
